# Association between type of birth attendants and neonatal mortality: Evidence from a National survey

**DOI:** 10.4314/ahs.v21i4.45

**Published:** 2021-12

**Authors:** Ololade Julius Baruwa, Acheampong Yaw Amoateng, Sibusiso Mkwananzi

**Affiliations:** 1 Africa Unit for Transdisciplinary Health Research, North-West University (Potchefstroom Campus) South Africa; 2 Population & Health Research Entity, Faculty of Humanities, North-West University (Mafikeng Campus) South Africa; 3 The Institute of Gender Studies, University of South Africa (UNISA)South Africa

**Keywords:** Neonatal, mortality, Lesotho

## Abstract

**Background:**

Although Lesotho has one of the highest childhood mortality levels in Southern Africa, there has been limited research on the link between type of birth attendant and neonatal mortality in Lesotho. This study examined the relationship between type of birth attendant and neonatal mortality while controlling for socio-demographic characteristics of mothers in Lesotho

**Methods:**

The study used data from the children's file of 2014 Lesotho Demographic and Health Survey data. Kaplan-Meier method was used to estimate neonatal mortality rate and Cox proportional hazard regression model was used to assess the association between type of birth attendant and neonatal mortality.

**Results:**

Result shows that 5.3% of all births attended to by non-SBAs resulted into neonatal mortality compared to 2.8% of those attended to by SBA. Result further shows that regardless of socio-demographic characteristics, the risks of neonatal mortality were significantly higher with non-SBAs compared to SBA in Lesotho (HR: 2.00, CI: 1.31–3.06).

**Conclusion:**

The risk of neonatal mortality is two times higher among children delivered by Non-SBA. Scale-up in access and uptake of SBA is recommended in Lesotho. Thus, Policy on scale-up access to SBA at delivery at no costs need to be put in place.

## Introduction

An integral part of the Sustainable Development Goal three (ensuring healthy lives and promoting the well-being at all ages) is to ensure universal access to sexual and reproductive healthcare services which include Skilled Birth Attendant (SBA) at delivery because access to skilled care at delivery is crucial to reducing maternal and child mortality^1^. According to the World Health Organization (WHO)^2^ definition, SBA is defined as any midwife, doctor or nurse who has been educated and trained to provide effective, uninterrupted and quality care in identifying, management and referral of complications in women and newborns during pregnancies, childbirth and the postnatal period. However, while the coverage of SBA has increased globally with 80% of live births occurring with the assistance of SBA, only about 50% of births were attended by SBA in sub-Saharan Africa^3^. Barriers mostly cited for not utilizing a skilled birth attendant includes; cost of transportation, high cost of care, and poor attitude of health workers among others^4^.

Studies that compare neonatal mortality by skilled birth attendants (SBA) and non-Skilled Birth Attendant (non-SBA) have suggested that the quality of the type of birth attendant is important for reducing the prevalence of neonatal mortality^5^,^6^. Other factors that have been found to be associated with neonatal mortality include age of woman^7^,^8^, place of residence^9^,^10^, marital status^11^,^12^, education and wealth^13^,^14^.

Studies have documented that neonatal mortality is expected to be reduced with births by SBA and increase with births by non-SBAs^5^,^15^,^16^. This is because non-SBAs are often associated with unhygienic cord care management, lack of disinfections of instruments used during delivery, lack of weighing of babies and improper management of birth injuries among others^5^,^17^. However, there has been limited research on effect of birth attendant on neonatal mortality in Lesotho.

Although there are limited information on neonatal mortality in Lesotho, data from the 2009 and 2014 Demographic and Health Survey (DHS) of Lesotho showed that neonatal mortality reduced from 47 deaths per 1000 live births to 35 deaths per 1000 live births in 2014. However, progress in the reduction of neonatal mortality remained slow in Lesotho. The aim of the present study therefore is to examine the effect of birth attendant type on neonatal mortality in Lesotho. We do this by also controlling for socio-demographic characteristics of mothers such as maternal age, maternal place of residence, maternal wealth index, maternal occupation, and maternal marital status.

## Methods

This study used a cross-sectional data, which was drawn from the children recode file of the 2014 Lesotho Demographic and Health Survey (LDHS). The LDHS was implemented by the Lesotho Ministry of Health (MOH), while technical assistance was provided by Inner City Fund (ICF) Macro through the MEASURE DHS program, a USAID-funded project. The sample for the 2014 LDHS was selected from a list of enumeration areas using the 2006 Lesotho Population and Housing Census (PHC) which was provided by the Lesotho Bureau of Statistics (BOS). Using probability proportional to size (PPS), 400 clusters of Enumeration Areas (EA) were drawn from the census sample frame, comprising of 118 and 282 clusters from urban and rural areas respectively.

The LDHS's children recode file contains information related to the child's pregnancy and birth, postnatal care and immunization and health of children of women born in the last five years preceding the survey. The data for the mother of each child is also included. This is because, children's information was collected from women aged 15–49 years (i.e. information about children were included in the woman's questionnaire). Information such as, sex of the child, month and year of birth of the child, child's survival status, age of child and age at death of child if the child had died among others.

### Ethical Approval

This research made used of DHS dataset, which is publicly available; however, mailed consent was provided to the authors as per DHS protocol. Detailed information regarding procedures and questionnaires are reported elsewhere http://www.dhsprogram.com/

### Variables

The outcome variable for the study is neonatal mortality which is measured as the death of a child during the first 28 days of life. The question of whether a child was dead or alive was answered by mothers in the survey. The child's survival status and the age at death in days are combined to generate the outcome variable and make it amenable to survival analysis. Specifically, children known to have died in the first 28 days of their lives are our interest in this study and are regarded as the event, while children who are still alive after 28 days at the time of the survey are treated as censored observations. The study population is made up of infants born to mothers who had live birth within five years preceding the survey.

The explanatory variable of interest in this study is birth attendant type which is dichotomized into SBA and non-SBA. SBA includes doctors, nurses and midwives, while non-SBA includes, traditional healers, relatives or friends, others, and by self. The socio-demographic variables we controlled for in the present study are variables that have been found to be associated with neonatal mortality from existing literatures 7. These control variables include maternal age (categorized as less than 25 years, 25–34 years and 35–49 years), maternal place of residence (categorized as urban and rural), maternal education (categorized as no education & primary education, and secondary & higher education), marital status (categorized as ever married and never married), and maternal wealth index (categorized as poor, middle and rich as opposed to the original measurement of poorest, poorer, middle, richer and richest from the DHS).

### Statistical Analysis

At the univariate level of analysis, a descriptive statistic using percentage distribution are used to describe the levels of neonatal mortality in Lesotho as well as all the predictor variables as reported by mothers. We also used the Kaplan-Meier curve to estimate neonatal mortality rate. For the multivariate analysis, the Cox proportional regression model was used to examine the effect of birth attendant type on neonatal death while controlling for the mother's socio-demographic variables. The assumption of the model is that the hazard ratio is constant over time and only covariates (such as education of mother, place of residence, and age of mother, marital status, occupation, and wealth index) that satisfy the assumption of the model are used. Stata 14 was used to analyzed the data and results are interpreted by using Hazard Ratio (HR) with level of significance set at p<0.05 and confidence intervals (CI) of 95%.

The Cox model is written as:

*h*(*t*) = *h*_0_ (*t*) × exp{*b*_1_*x*_1_ + *b*_2_*x*_2_ +...+ *b_p_x_p_*}

Where the hazard function *h*(*t*) is the dependent variable, which is dependent on a set of p covariate

(*x*_1_,*x*_2_,..., *x_p_* whose impact is measured by the size of the respective coefficients. (*b*_1_, *b*_2_,..., *b_p_*. The term *h*_0_ is the baseline hazard, which gives the value of the hazard if all the *x_i_* are equal to zero.

## Results

Table 1 shows that 3.5% of children born five years preceding the survey died in their first month of birth. The distribution mother's background characteristic is also presented in Table 1. Most (74.9%) of the mothers interviewed lived in rural areas while one-quarter of the mothers (25.1%) lived in urban areas. Half (50.3%) of the mothers had more than primary education and also about half (46.6.7%) proportion of them are from a poor household wealth index. Slightly, over one-fifth (20.3%) of the mothers are from middle wealth households. The largest (44.4%) of the age-group of mothers were between the ages of 25–34 years while 17.1% were between the ages of 35–49. Most (88.9%) of the mothers interviewed were employed and many (88.9%) were previously or currently married as at the time of the survey.

**Table 1:** Percentage distribution of socio-demographic characteristics of neonates' mothers

Variables	Frequency (N)	Percentage (%)
**Neonatal death** No Yes	3029 109	96.5 3.5
**Birth attendant types** Skilled birth attendant Non-skilled birth attendant	2371 760	75.7 24.3
**Place of residence** Urban Rural	786 2352	25.1 74.9
**Education** No education or primary Secondary or higher	1558 1580	49.7 50.3
**Wealth index** Poor Middle Rich	1461 636 1041	46.6 20.3 33.7
**Age** <25 25–34 35–49	1207 1394 537	38.5 44.4 17.1
**Marital status** never married ever married	347 2791	11.1 88.9

### The Bivariate Analysis

Table 2 shows the prevalence and Chi-square analysis of neonatal mortality by type of birth attendant type and mother's socio-demographic characteristics. The table shows that there is a statistical relationship between birth attendant types and neonatal mortality. Result show that 5.3% of all births attended to by non-SBAs resulted into neonatal mortality.

**Table 2:** Bivariate result showing neonatal mortality by selected characteristics of mothers

	Neonatal mortality		
	No (%)	Yes (%)	P-value (X^2^)
**Variable**			
**Birth attendant type** Skilled birth attendant Non-skilled birth attendant	2305 (97.2) 720 (94.7)	66 (2.8) 40 (5.3)	0.01 (10.82)
**Place of residence** Urban Rural	765 (97.3) 2264 (96.3)	21 (2.7) 88 (3.7)	0.16 (2.01)
**Education** No education or primary Secondary or higher	1502 (96.4) 1527 (96.5)	56 (3.6) 53 (3.4)	0.71 (0.13)
**Wealth index** Poor Middle Rich	1408 (96.4) 616 (96.5) 1005 (96.5)	53 (3.6) 20 (3.1) 36 (3.5)	0.86 (0.31)
**Age** <25 25–34 35–49	1168 (96.8) 1341 (96.2) 520 (96.8)	39 (3.2) 53 (3.8) 17 (3.2)	0.67 (0.81)
**Marital status** Never married Ever married	340 (98.0) 2689 (96.5)`	7 (2.0) 102 (3.5)	0.12 (2.47)

Chi-square test analysis*

All the socio-demographic variables used show no significant relationship, but the prevalence of neonatal mortality varies across all socio-demographic variables. Table 2 shows that neonatal mortality is 3.7% in rural areas, 3.6% among neonates whose mother have no education or primary education and 3.6% among neonates born to mothers that belong to poor household wealth index. Neonatal mortality is 3.8% among mothers that are between the ages of 25–34 years and 2% among children of mothers that are never married.

**Figure 1:**
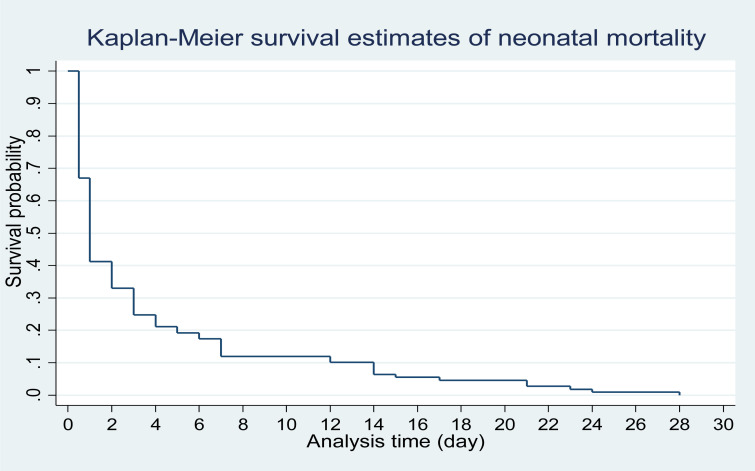
K-M plot showing the estimate of neonatal mortality in Lesotho

The graph in Figure 1 shows the Kaplan-Meier survival estimates for time to neonatal mortality from day 0 to day 28 among all children (109) that died in the first 28 days of life. The figure shows that about 33% of neonatal mortality occurred on the day of birth (day 0) and by day one, more than half (approximately 59%) of neonatal mortality had occurred. A plausible explanation could be that most of the neonatal mortality in Lesotho occurred as a result of complications from delivery at birth, which might result from complications from birth and/or unskilled birth attendant.

### Multivariate analysis

The results of the multivariate analysis are presented in Table 3. The unadjusted hazard of neonatal mortality is significantly higher among children born to mothers who use non-SBA (HR: 1.9, CI =1.29–2.83). After controlling for place of residence, education, wealth index, and marital status, the hazard of neonatal mortality remained significantly higher among children born to mothers who used non-SBA compared to children born to mothers who use SBA (HR: 2.00, CI =1.31–3.06). In fact, children born to mothers who used non-SBA attendants are twice more likely to die in their first 28 days compared to children born to mothers who use medical birth SBA.

**Table 3:** Multivariate analysis showing the effect of birth attendant types on neonatal mortality while controlling for socio-demographic variables of mothers

Variable	Unadjusted model	Adjusted model
	HR (95% CI)	HR (95% CI)
**Birth Attendant** Skilled birth attendant Non-skilled birth attendant	1 1.91 (1.29–2.83*)	1 2.00 (1.31–3.06*)
**Place of Residence** Urban Rural	1 1.40 (0.87–2.26)	1 1.55 (0.89–2.78)
**Education** No education/primary Secondary/ higher	1 0.93 (0.64–1.36)	1 1.11 (0.71–1.73)
**Wealth index** Poor Middle Rich	1 0.87 (0.52–1.45) 0.95 (0.63–1.46)	1 1.04 (0.60–1.82) 1.43 (0.82–2.50)
**Age** <25 25–34 35–49	1 1.18 (0.82–2.50) 0.98 (0.55–1.73)	1 1.10 (0.72–1.70) 0.88 (0.49–1.58)
**Marital Status** Never married Ever married	1 1.82 (0.85–3.91)	1 1.69 (0.78–3.69)

* denotes a significant result.

Maternal age has no relationship with neonatal mortality in both the unadjusted and adjusted analysis as shown in Table 3. Similarly, there is no significant association between neonatal mortality and other socio-demographic variables such as place of residence, mother's education, wealth index and marital status.

## Discussion

The aim of the present study was to examine the effect of birth attendant types on neonatal mortality in Lesotho, controlling for socio-demographic characteristics of the mothers. This study found that of 3138 children born to mothers in the 2014 Lesotho DHS, 3.5% died at the neonate stage. A finding which shows that the neonatal mortality rate is 34.7 deaths per 1000 live births. This finding is a quite different from 45 deaths per 1000 live birth that was estimated by the World Health Organization in 2012. The reduction in the rate of neonatal mortality suggests that various interventions and programs adopted by the government of Lesotho and other non-government organization have yielded a significant but slow progress in the reduction of neonatal mortality in Lesotho.

The results of the multivariate analysis have shown that the type of birth attendant used by mothers is the main predictor of neonatal mortality in Lesotho. Specifically, we found that neonatal mortality is higher among children delivered by non-SBA. The multivariate analysis showed that none of the socio-demographic characteristics of mother was associated with neonatal mortality. However, it is important to investigate further using a qualitative study. The finding that non-SBAs increase the risk of neonatal mortality is consistent with previous studies on the same subject^5^,^17^. Moreover, this finding is consistent with Mosley and Chen's child survival theory which underscores personal illness control, personal preventive measures and medical treatment of mothers as some of the proximate determinants of child survival^18^.

The practices and the quality of care during pregnancy and after childbirth are important factors for child survival. Thus, the fact that non-SBA is often characterized by unhygienic cord care, lack of disinfections of instruments used during delivery, lack of weighing of babies and improper management of birth outcomes such as birth injuries, preterm birth and low birth weight among others may be the reason for higher rate of neonatal mortality among children delivered by non-SBA^17^,^19^. The finding of this present study suggests that lack of proper management of birth outcomes during delivery by non-SBAs may increase neonatal mortality. Also, the absence of important health facilities to help manage complications during and after delivery may increase the risk of neonatal mortality among children delivered by non-SBAs.

While the present study sought to make a contribution to the existing body of knowledge on child mortality, some main limitations should be considered while interpreting the results of the study. Firstly, even though the Demographic and Health Surveys are reputable worldwide, the data of these surveys are based on information provided by human beings, in this instance, mothers whose responses could be affected by both social desirability concerns and recall biases. For instance, some mothers may not feel comfortable reporting the death of their infants. Likewise, a long recall of an event such as death might introduce a recall bias. However, this limitation was minimized by restricting the study population to the most recent deliveries 5 years before the survey. Secondly, there might be misclassification of neonatal mortality and still birth by the respondents.

## Conclusion

The study found that neonatal mortality is higher among children delivered by non-SBAs. However, all maternal socio-demographic factors that have been largely explored were not significantly associated with neonatal mortality in Lesotho. The very fact that the risk of neonatal mortality is doubled with non-SBA has underscored the need for more skilled birth attendants that can manage the many complications that accompany such deliveries. Thus, it is important for the Government to deploy skilled birth attendants to areas that lack skilled birth attendant, as well as increasing the numbers of SBAs where necessary. Further, there is a need for community-based educational programs to sensitize communities on the importance of safe delivery practices by delivering with SBAs. Policy on scale-up access to SBA at delivery at no costs need to be put in place.
